# *Ficus deltoidea*: A Potential Alternative Medicine for Diabetes Mellitus

**DOI:** 10.1155/2012/632763

**Published:** 2012-06-03

**Authors:** Zainah Adam, Shafii Khamis, Amin Ismail, Muhajir Hamid

**Affiliations:** ^1^Medical Technology Division, Malaysian Nuclear Agency, Bangi, Selangor 43000 Kajang, Malaysia; ^2^Department of Nutrition and Dietetics, Faculty of Medicine and Health Sciences, Universiti Putra Malaysia, Selangor 43400 Serdang, Malaysia; ^3^Department of Microbiology, Faculty of Biotechnology and Biomolecular Sciences, Universiti Putra Malaysia, Selangor 43400 Serdang, Malaysia

## Abstract

*Ficus deltoidea* from the Moraceae family has been scientifically proven to reduce hyperglycemia at different prandial states. In this study, we evaluate the mechanisms that underlie antihyperglycemic action of *Ficus deltoidea*. The results had shown that hot aqueous extract of *Ficus deltoidea* stimulated insulin secretion significantly with the highest magnitude of stimulation was 7.31-fold (*P* < 0.001). The insulin secretory actions of the hot aqueous extract involved K^+^_ATP_ channel-dependent and K^+^_ATP_-channel-independent pathway. The extract also has the ability to induce the usage of intracellular Ca^2+^ to trigger insulin release. The ethanolic and methanolic extracts enhanced basal and insulin-mediated glucose uptake into adipocytes cells. The extracts possess either insulin-mimetic or insulin-sensitizing property or combination of both properties during enhancing glucose uptake into such cells. Meanwhile, the hot aqueous and methanolic extracts augmented basal and insulin-stimulated adiponectin secretion from adipocytes cells. From this study, it is suggested that *Ficus deltoidea* has the potential to be developed as future oral antidiabetic agent.

## 1. Introduction

Type 2 diabetes mellitus is characterized by persistent hyperglycemia resulting from defect of insulin secretion and insulin action [1]. Long-term hyperglycemia is a major factor in development of pathogenesis of diabetic complications [2, 3]. Currently, type 2 diabetes mellitus is treated with antidiabetic agents such as sulphonylureas, meglitinides, thiazolidinediones groups, and so forth. Although plenty of antidiabetic agents are available, prevalence of the disease remains major global health problem [4]. This could possibly be due to the limitations of these agents such as undesirable side effects [5]. For instance, sulphonylureas treatment was associated with hypoglycemia and weight gain [6–8]. Meglitinides treatment causes hypoglycemia, rhinitis, bronchitis, and headache [9]. Meanwhile, thiazolidinediones can cause fluid retention, weight gain, anemia, and liver injury [10, 11]. These limitations have fueled the search for new antidiabetic drugs for treatment of diabetes mellitus.

Stimulation of insulin secretion from pancreatic *β* cells is one of the mechanisms by which antidiabetic agents reduce hyperglycemia [12, 13]. The secreted insulin then facilitates glucose uptake in insulin-sensitive cells such as muscle, liver, and adipocytes, hence reduces hyperglycemia [14]. Besides, augmentation of adiponectin secretion from adipocytes cells also has been well accepted as antidiabetic mechanism. This adipocyte-derived factor has been reported to improve insulin sensitivity in skeletal muscle and liver [15–17] resulting in stimulation of glucose utilization and fatty acid oxidation [18], enhancement of glucose uptake through the increment of expression and translocation of GLUT4 [19], suppression of gluconeogenesis in the liver [20] and enhancement of insulin signaling in skeletal muscle [21].

*Ficus deltoidea*, from the Moraceae family, is one of the common medicinal plants in Malaysia. It has been used to relieve headache, fever, and toothache. Decoction of the whole plant has been used as herbal drink by women after birth to strengthen the uterus [22, 23]. Based on ethnobotanical approaches, this plant has been claimed to have antidiabetic properties [22]. Few scientific studies were done and confirmed the antidiabetic activity of this plant. Acute treatment of* F. deltoidea *extracts reduced hyperglycemia in normal and diabetic rats at different prandial states [24–26]. Following 15-day treatment, hot aqueous extract of* F. deltoidea *stimulated insulin release and reduced fasting hyperglycemia [27]. Elucidation of antihyperglycemic mechanisms demonstrated that this plant enhanced basal and insulin-stimulated glucose uptake into liver cells [28] and reduced the rate of glucose absorption from small intestine by inhibiting intestine sucrase activity [29]. The present study was performed to find other possible antidiabetic mechanisms of *F. deltoidea*, if any, by evaluating the potential of this plant to stimulate insulin secretion from pancreatic *β* cells, to enhance glucose uptake into adipocyte cells, and to augment adiponectin secretion from adipocyte cells.

## 2. Methodology

### 2.1. Chemicals and Reagents

BRIN BD11 cell line was a gift from the Animal Cell Culture Laboratory, Faculty of Biotechnology and Biomolecular Sciences, Universiti Putra Malaysia. 3T3F442A preadipocytes were purchased from the European Collection of Cell Cultures (ECACC, Salisbury, UK). All cell culture supplements were purchased from Invitrogen, USA. Ethanol and methanol were purchased from J. T. Baker Chemical Co. Sodium chloride (NaCl), potassium chloride (KCl), calcium chloride (CaCl_2_), potassium dihydrogen phosphate (KH_2_PO_4_), magnesium sulphate (MgSO_4_), sodium hydrogen carbonate (NaHCO_3_), HEPES, sodium dodecyl sulphate (SDS), 3-(4,5-dimethylthiazol-2-yl)-2,5-diphenyltetrazolium bromide (MTT), bovine insulin, ammonium hydroxide (NH_4_OH), dimethylsulphoxide (DMSO), glibenclamide, isobutylmethylxanthine (IBMX), tolbutamide, diazoxide, verapamil, and D-glucose were purchased from Sigma Chemical Co. (St. Louise, MO, USA). Ultima Gold LLT was purchased from PerkinElmer (USA). 2-Deoxy-[1-^3^H]-glucose was purchased from GE Healthcare (USA). Rosiglitazone maleate (Avandia) was purchased from a local pharmacy.

### 2.2. Plant Materials and Extraction Procedure

Plants of *F. deltoidea *were collected at the Sungai Tengi Selatan, Selangor, Malaysia. The plants were identified by a taxonomist from the Biodiversity Unit of the Institute of Bioscience, Universiti Putra Malaysia. A specimen (SK1467/07) was deposited at the herbarium of the above institute. The leaves of *F. deltoidea *were oven dried at 45°C and ground to a fine powder. Hot aqueous extracts were prepared by boiling the powdered sample in distilled water for 3 hours (100 g/L) by changing the water every hour. The combined suspension was filtered using Whatman filter paper no. 54 and freeze-dried to give the powdered form. Ethanolic extract was prepared by soaking the powder in 95% ethanol for 3 days (100 g/L) at room temperature by changing solvent daily. The combined suspension was filtered using Whatman filter paper no. 54 and evaporated to dryness under pressure at 30°C to give an ethanolic extract. Methanolic extract was prepared by soaking the powder in 95% methanol for 3 days (100 g/L) at room temperature by changing solvent daily. The combined suspension was filtered using Whatman filter paper no. 54 and evaporated to dryness under pressure at 30°C to give a methanolic extract. The yield of the extract was found to be 21 g, 17 g, and 19 g for hot aqueous, ethanolic, and methanolic extract, respectively.

### 2.3. Cell Viability Assay

The viability of all cell lines in the presence of *F. deltoidea* hot aqueous, ethanolic, and methanolic extracts was conducted according to the method of Mosmann [30] and Carmichael et al. [31]. BRIN BD11 cell line was maintained in the Roswell Park Memorial Institute (RPMI) 1640 medium and 3T3F442A preadipocytes were maintained in Dulbecco's modified Eagle's medium (DMEM). Complete culture medium was supplemented with 10% (v/v) foetal bovine serum (FBS) and 1% (v/v) antibiotic solution (10,000 units/mL penicillin and 10 mg/mL streptomycin) at 37°C humidified with 5% CO_2_. The confluent cells were seeded at concentration of 1.5 × 10^5^ cells/well onto a sterile 96-well plate and incubated at 37°C overnight. Cells were further incubated at 37°C for 72 hours in the absence or presence of *F. deltoidea* extracts (10–1000 *μ*g/mL) and glibenclamide (10–2000 *μ*M). Following the required incubation period, 20 *μ*L of 5 mg/mL of 3-(4,5-dimethylthiazol-2-yl)-2,5-diphenyltetrazolium bromide (MTT) was added to each well and incubated for 4 hours. Subsequently, the media from each well was then gently aspirated and 100 *μ*L of dimethylsulphoxide (DMSO) was added to dissolve the formazan crystals. Plates were shaken for 5 seconds, and absorbance was measured at 570 nm using the Anthos microplate reader (Beckman Coulter, USA). 

### 2.4. Insulin Secretion Assay

Insulin secreting activity of *F. deltoidea* was evaluated using BRIN-BD11 cells. The generation and basic characteristics of this glucose-responsive insulin-secreting cell line have been described elsewhere [32]. BRIN BD11 cell line was maintained in RPMI 1640 medium supplemented with 10% (v/v) FBS and 1% (v/v) antibiotic solution (10,000 units/mL penicillin and 10 mg/mL streptomycin) at 37°C humidified with 5% CO_2_. Insulin secretion assay was conducted according to the method of Gray and Flatt [33] with brief modifications. Cells were seeded at a concentration of 2.5 × 10^5^ cells/well in a 12-well plate and incubated at 37°C overnight to allow attachment prior to test. The next day, cells were washed thrice with the Krebs-Ringer bicarbonate buffer (KRB) and preincubated with this KRB for 40 minutes at 37°C. Cells were further incubated for 1 hours with KRB (negative control) KRB containing *F. deltoidea* hot aqueous, ethanolic, and methanolic extracts (10–1000 *μ*g/mL) KRB containing glibenclamide (10–2000 *μ*M). All evaluations were performed at 2 mM glucose. Insulin concentration was determined using Rat Insulin ELISA Kits (Mercodia AB, Sweden).

### 2.5. Elucidation of Insulin Secretion Mechanisms

*F. deltoidea *extracts that showed tremendous insulin secretion activity were further evaluated for determination of their insulin secretory mechanisms. The evaluations were conducted according to the method of Hannan et al. [34]. Extracts were incubated with modulators that are known to affect secondary messenger pathway in pancreatic *β*-cells such as insulin secretagogue (isobutylmethylxanthine (IBMX) and tolbutamide), insulin secretion inhibitor (diazoxide and verapamil), and potassium chloride (KCl) at depolarizing concentration. Cells were seeded at a concentration of 2.5 × 10^5^ cells/well in a 12-well plates and incubated overnight at 37°C to allow attachment prior to test. The next day, cells were washed thrice with KRB and preincubated with KRB for 40 minutes at 37°C. Further incubation was done for 1 hour with KRB containing *F. deltoidea* extract in the absence and presence of 100 *μ*M of IBMX, 200 *μ*M of tolbutamide, 300 *μ*M of diazoxide, 50 mM of verapamil, and 30 mM of KCl. All evaluations were performed at 2 mM glucose. Insulin concentration was determined using Rat Insulin ELISA Kits (Mercodia AB, Sweden).

### 2.6. Glucose Uptake Assay

3T3F442A preadipocytes was maintained in DMEM supplemented with 10% (v/v) FBS and 1% (v/v) antibiotic solution (10,000 units/mL penicillin and 10 mg/mL streptomycin) at 37°C humidified with 5% CO_2_. The 3T3F442A preadipocytes were differentiated into adipocytes spontaneously when reach confluency. At this time culture medium was supplemented with 5 *μ*g/mL insulin. Glucose uptake assay was done according to Liu et al. [35] with some modifications. Briefly, confluent cells were seeded at a concentration of 2 × 10^5^ cells/well in a 12-well plate and left overnight at 37°C to allow attachment prior to test. The next day, cells were washed thrice with serum-free DMEM and preincubated with this medium for 2 hours at 37°C. After starvation period, cells were washed thrice with the Krebs-Ringer bicarbonate buffer (KRB). Cells were further incubated for 30 minutes at 37°C with various concentrations of *F. deltoidea* extracts (10–1000 *μ*g/mL) either alone or in combination with 100 nM insulin. Rosiglitazone maleate (Avandia) was used as positive control. To initiate glucose uptake reaction, 2-deoxy-[1-^3^H]-glucose (1 *μ*Ci/mL) diluted in 0.1 mM D-glucose solution was added to each well and incubated further for 60 minutes at 37°C. After incubation, cells were washed thrice with ice-cold KRB buffer and solubilized with 0.1% sodium deodecyl sulphate (SDS) dissolved in phosphate buffer, pH 7.4. The content of each well was transferred into scintillation vials, and 15 mL of scintillation cocktail, Ultima Gold LLT, was added. The radioactivity incorporated into the cells was measured using liquid scintillation counter (Hewlett Packard, USA).

### 2.7. Adiponectin Secretion Assay

The potential of *F. deltoidea* to stimulate adiponectin secretion was evaluated in 3T3F442A adipocyte cells. Adiponectin secretion assay was conducted according to the method of Roffey et al. [36], with brief modifications. Confluent cells were seeded at a concentration of 2 × 10^5^ cells/well in 12-well plate and left overnight at 37°C humidified with 5% CO_2_ to allow attachment prior to test. The next day, cells were washed thrice with serum-free DMEM and pre-incubated with this medium for 2 hours at 37°C. After starvation period, cells were washed thrice with the Krebs-Ringer bicarbonate buffer (KRB). Cells were further incubated for 60 minutes at 37°C with various concentrations of *F. deltoidea* extracts (10–1000 *μ*g/mL) either alone or in combination with 100 nM insulin. Rosiglitazone maleate was used as positive controls. Adiponectin secreted in the solution was measured using Rat Adiponectin ELISA Assay Kits (BioVision, USA).

### 2.8. Statistical Analysis

Results are expressed as mean ± standard deviation for a given number of observations. Statistical analyses were performed using GraphPad Prism Software version 4.0 (serial no. GpW4-015977-RHB-3678). The data were analyzed using one-way analysis of variance (ANOVA), followed by Tukey's post hoc test. The group means were considered significantly different at the level of *P* < 0.05.

## 3. Results

### 3.1. Cells Viability in the Presence of *F. deltoidea* Extracts

The effects of *F. deltoidea* extracts and glibenclamide on BRIN BD11 cell viability are shown in Table 1. All extracts' and glibenclamide, at particular, concentrations reduced BRIN BD11 cells viability significantly. Ethanolic and methanolic extracts at concentrations of 500 and 1000 *μ*g/mL as well as glibenclamide at concentration of 2000 *μ*M reduced the viability of BRIN BD11 cells to less than 50%. In 3T3F442A adipocytes, all *F*.* deltoidea* extracts reduced cell viability significantly at all concentrations evaluated (Table 2). However, no extract reduced cell viability to less than 50%. Rosiglitazone maleate exhibited a reduction in cell viability at concentration 7 to 140 *μ*M, and it was shown that reduction at the highest concentration was more than 50%.

### 3.2. Insulin Secreting Activity of *F. deltoidea* Extracts

**Figure 1:**
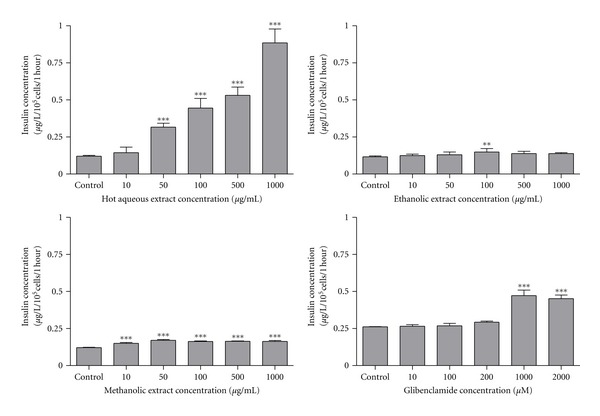
Effect of *F. deltoidea* extracts and glibenclamide on insulin secretion activity from BRIN BD11 cells. Values are expressed as mean ± standard deviation (*n* = 4 to 8). **P < 0.01 and ***P < 0.01 compared with control.

Effects of *F. deltoidea* extracts and glibenclamide on insulin secretion from BRIN BD11 cells are shown in Figure 1. Hot aqueous extract exhibited a stepwise stimulatory effect on insulin secretion at 2 mM glucose. Significant stimulation was observed at concentrations 50, 100, 500, and 1000 *μ*g/mL which evoked a 2.61-(*P* < 0.001), 3.68-(*P* < 0.001), 4.38-(*P* < 0.001) and 7.31-(*P* < 0.001) fold of stimulation, respectively, compared to control. In the presence of ethanolic extract, a significant stimulation of insulin secretion (1.27-fold; *P* < 0.001) was only observed at concentration 100 *μ*g/mL. Meanwhile, the methanolic extract significantly stimulated insulin secretion at all concentrations evaluated with the magnitude of insulin secretion 1.24-fold (*P* < 0.001), 1.41-fold (*P* < 0.001), 1.34-fold (*P* < 0.001), 1.36-fold (*P* < 0.001), and 1.36-fold (*P* < 0.001) obtained at concentrations of 10, 50, 100, 500, and 1000 *μ*g/mL, respectively. Glibenclamide had significantly stimulated insulin secretion at concentrations of 1000 *μ*M (1.80-fold; *P* < 0.001) and 2000 *μ*M (1.73-fold; *P* < 0.001). The former concentration was used as standard reference because it shows the highest magnitude of stimulation following a dose-response evaluation.

### 3.3. Insulin Secretion Mechanisms of Hot Aqueous Extract

**Figure 2:**
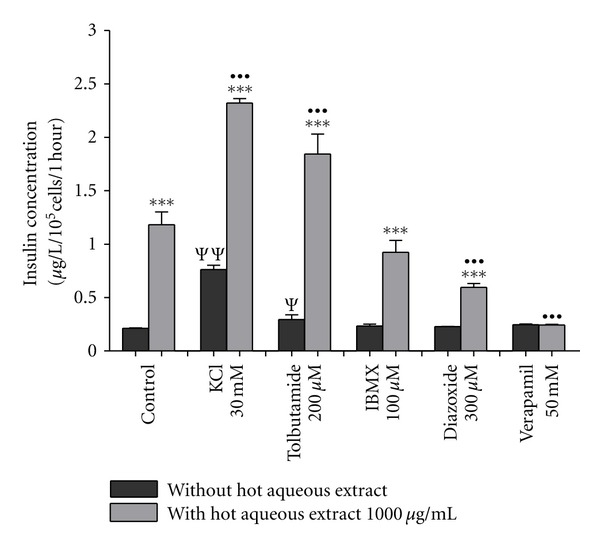
The influence of insulin secretion modulators on the effect of 1000 *μ*g/mL hot aqueous extract on insulin secretion from BRIN BD11 cells. Values are expressed as mean ± standard deviation (*n* = 4). ***P < 0.001 compared with incubation without hot aqueous extract in the respective treatment group. ^•••^ *P* < 0.001 compared with control treated with hot aqueous extract. ^Ψ^ *P* < 0.05; ^ΨΨ^ *P* < 0.01 compared with untreated control.

The influence of insulin secretion modulators on the effect of 1000 *μ*g/mL hot aqueous extract on insulin secretion from BRIN BD11 cells is shown in Figure 2. All evaluations were done at basal glucose concentration (2 mM). It was shown that 1000 *μ*g/mL of hot aqueous extract significantly stimulated basal insulin secretion (untreated control) by 5.57-fold (*P* < 0.001). All modulators showed no effect on basal insulin secretion except 30 mM KCl which stimulated basal insulin secretion by 3.60-fold (*P* < 0.001). Insulin-releasing effect of 1000 *μ*g/mL of hot aqueous was potentiated in the presence of all modulators except for 50 mM verapamil which exhibited similar magnitude of secretion between the absence and presence of 1000 *μ*g/mL of hot aqueous extract. The magnitudes of potentiation were 3.04-fold (*P* < 0.001), 6.30-fold (*P* < 0.001), 3.95-fold (*P* < 0.001), and 2.60-fold (*P* < 0.001) in the presence of 30 mM of KCl, 200 *μ*M of tolbutamide, 100 *μ*M of IBMX, and 300 *μ*M of diazoxide, respectively. Combinations of 1000 *μ*g/mL of hot aqueous extract with 30 mM KCl and tolbutamide had escalated the insulin-releasing effect of the extract when compared to the extract alone. The magnitude of escalations were 1.96-fold (*P* < 0.001) and 1.56-fold (*P* < 0.001), respectively. In contrast, combinations of 1000 *μ*g/mL of hot aqueous extract with diazoxide and verapamil had inhibited insulin-releasing effect of the extract with magnitudes of inhibition 0.49-fold (*P* < 0.001) and 0.79-fold (*P* < 0.001), respectively. Meanwhile the IBMX has no effect on insulin secretion causes by 1000 *μ*g/mL of hot aqueous extract.

### 3.4. Glucose Uptake Activity of *F. deltoidea* Extracts

Table 3 shows the effects of *F. deltoidea* extracts on glucose uptake activity in 3T3F442A adipocytes. Insulin (100 nM) alone enhanced glucose uptake by 1.55-fold (*P* < 0.001) relative to control. Hot aqueous extract has no effect on glucose uptake activity in 3T3F442A adipocytes. Ethanolic extract increased basal and insulin-mediated glucose uptake in a concentration-dependent manner. Basal glucose uptake was enhanced at concentrations of 500, and 1000 *μ*g/mL. Enhancement effect produced by the later concentration was 1.32-fold (*P* < 0.01) higher than that of 100 nM insulin alone. Insulin-mediated glucose uptake was enhanced at concentrations of 100, 500, and 1000 *μ*g/mL. Enhancement effect produced by the highest concentration was 1.29-fold (*P* < 0.05) higher than that of 100 nM insulin. The methanolic extract also exhibited concentration-dependent enhancement of glucose uptake. Basal uptake was significantly enhanced at concentrations of 500 and 1000 *μ*g/mL. The enhancement by the latter concentration was 1.27-fold (*P* < 0.05) higher than that of 100 nM insulin alone. Insulin-mediated uptake was significantly enhanced at concentrations of 100, 500, and 1000 *μ*g/mL. Rosiglitazone maleate at concentrations of 35 *μ*M and 70 *μ*M was used as positive control to challenge the effects of *F. deltoidea* on basal and insulin-mediated glucose uptake in 3T3F442A adipocytes, respectively. These concentrations were chosen because they shows highest magnitude of enhancement following a dose-response evaluation.

### 3.5. Adiponectin Secreting Activity of *F. deltoidea* Extracts

Table 4 shows the effect of *F. deltoidea* extracts on adiponectin secretion from 3T3F442A adipocytes. 100 nM insulin stimulated adiponectin secretion by 1.64-fold relative to control. Hot aqueous extract augmented adiponectin secretion in a concentration-dependent manner. Basal adiponectin secretion was significantly increased at concentrations of 500 and 1000 *μ*g/mL, respectively. Increments of adiponectin secretion by such concentrations were 1.26-fold (*P* < 0.001) and 1.56-fold (*P* < 0.001) higher than that of 100 nM insulin alone, respectively. Likewise, insulin-stimulated adiponectin secretion was significantly increased at concentrations of 500 and 1000 *μ*g/mL. The increment effect by the latter concentration was 1.27-fold higher than that of 100 nM insulin alone. For both concentrations of the extracts, there were no differences in adiponectin secretion between basal and insulin-stimulated state. Meanwhile, the ethanolic extract of *F. deltoidea* has no effect on adiponectin secretion from 3T3F442A adipocytes. Similar to hot aqueous extract, the methanolic extract increased adiponectin secretion in a concentration-dependent manner. Basal adiponectin secretion was significantly increased at concentrations of 500, and 1000 *μ*g/mL. Under insulin-stimulated state, methanolic extract at concentrations of 10, 50, 100, 500 and 1000 *μ*g/mL increased adiponectin secretion. Rosiglitazone maleate at concentrations of 70 *μ*M and 35 *μ*M was used as positive controls to challenge effect of *F. deltoidea* extracts on basal and insulin-stimulated adiponectin secretion, respectively. These concentrations were chosen because they produces the highest stimulation effect after a dose response evaluation. The enhancement of adiponectin secretion by *F. deltoidea* hot aqueous and methanolic extracts was lesser than that of the positive controls.

## 4. Discussions

The present study reports for the first time the insulin secreting, glucose uptake, and adiponectin secretion properties of *F. deltoidea* plant. These suggests that there is possibility of presence of antidiabetic compounds in the *F. deltoidea* plant which exert antidiabetic action through stimulation of insulin secretion from pancreatic *β* cells, enhancement of glucose uptake by adipocytes cells, and stimulation of adiponectin secretion in adipocytes cells. Previous research had reported that flavonoids stimulated insulin secretion from pancreatic *β* cells [37], stimulated adiponectin secretion in adipocytes cells [16], and enhanced glucose uptake into adipocytes cells in an insulin-mimetic manner [38]. In addition, *Eucalyptus globules *plants which possess insulin secreting activity have been reported to contain flavonoids and tannins [39]. Phytochemical screening of *F. deltoidea* revealed the presence of high amount of flavonoids and tannins [40]. Thus, there is possibility that these secondary metabolites account for the observed pharmacologic effects of the *F. deltoidea* plant.

The viability of BRIN BD11 cell in the presence of *F. deltoidea* extracts at various concentrations was evaluated using MTT assay. In this assay, the yellow tetrazolium salt, MTT, is reduced by the mitochondrial enzymes, succinate dehydrogenase, to form insoluble purple formazan crystals which are solubilized by the addition of a detergent [30]. The color produced then can be measured spectrophotometrically at 570 nm [31]. MTT reduction was proportional to cell viability [30]. Following 72-hour treatment, all extracts at particular concentrations reduced BRIN BD11 and 3T3F442A adipocyte cells significantly. According to Elmore et al. [41], the highest concentration of a test agent in cytotoxicity evaluation should be 1000 *μ*g/mL or 1000 *μ*M. If none of the concentrations of test agents exhibited cytotoxic effect in excess of 50% of cell populations, the test agent is considered nontoxic against the tested cell line. In the present study, ethanolic and methanolic extracts at concentrations of 500 and 1000 *μ*g/mL reduce the viability of BRIN BD11 cells to less than 50%. Thus, both extracts were considered toxic against BRIN BD11 cells [41].

Insulin is an anabolic hormone secreted by pancreatic *β* cells in response to elevation of blood glucose concentration especially after meal [42, 43]. This hormone maintains body glucose homeostasis by regulating metabolism of carbohydrate, lipid, and protein [44]. Insulin reduced hyperglycemia by facilitating glucose disposal from blood circulation into insulin-responsive cells such as muscle, adipose, and liver [45, 46]. The secretion of insulin involved two major signaling pathways, K^+^_ATP_ channel-dependent and K^+^_ATP_ channel-independent pathways [42, 47]. The former is responsible for the first-phase glucose-stimulated insulin secretion whereas combination of both pathways responsible for second-phase secretion [48]. The underlying mechanisms involved in the K^+^_ATP_ channel-dependent pathway are well defined. In this pathway, glucose is transported into *β* cells through facilitated diffusion of GLUT2 glucose transporters and this glucose undergoes metabolism to produce ATP molecules. The increase of intracellular ATP/ADP ratio results in the closure of the K^+^_ATP_ channel and leads to depolarization of the membrane. This causes the opening of voltage-dependent calcium (Ca^2+^) channels and facilitating extracellular Ca^2+^ influx into the *β* cell. A rise in free cytosolic Ca^2+^ eventually activates an effectors system responsible for translocation of insulin-containing secretory granules to the plasma membrane and the exocytotic release of insulin [43, 48]. Meanwhile, mechanisms underlying the K^+^_ATP_ channel-independent pathway remain unclear. Two mechanisms have been suggested; the enhancement of the stimulatory effect of Ca^2+^ on the secretory process [43] and the changes of regulators of the K^+^_ATP_ channel-independent pathway, adenine nucleotide concentration [42].

In the present study, hot aqueous extract of *F. deltoidea *stimulates insulin secretion significantly with the highest magnitude of stimulation 7.31-fold (*P* < 0.001) obtained at concentration of 1000 *μ*g/mL. This stimulation was greater than that of 1000 *μ*M glibenclamide. This suggests that *F. deltoidea *may contain water-soluble insulin-secreting compounds that are more potent than glibenclamide. Viability study had confirmed that hot aqueous extract did not influence BRIN BD11 cells. These observations suggest that the hot aqueous extract of *F. deltoidea *has the enormous potential to be developed as new oral hypoglycemic agents targeted on stimulation of insulin secretion. Ethanolic extract (100 *μ*g/mL) and methanolic extract (all concentrations) induced an increased insulin secretion. However, both extracts were found toxic against BRIN BD11 cells. This could possibly be due to the presence of toxic compounds in both extracts which can damages subcellular organelles of the cells and lead to cell death [49]. Thus, the efficacy of ethanolic and methanolic extracts in stimulating insulin secretion may not be taken into account.

The 1000 *μ*g/mL of hot aqueous extract was further evaluated for determination of mechanisms underlying its insulin secretory action. This concentration of extract was chosen because it possesses the highest insulin secretion property among all *F. deltoidea* extracts. The extract was incubated in the presence and absence of insulin secretion modulators such as the insulinotropic agonist (tolbutamide and IBMX), insulinotropic antagonist (diazoxide and verapamil), and depolarization concentration of KCl (30 mM). It was shown that 30 mM KCl stimulated basal insulin secretion whereas other modulators did not. This could possibly be due to that 30 mM KCl fully depolarized the plasma membrane, hence activate Ca^2+^ channel and trigger insulin release [50, 51]. Tolbutamide, a hypoglycemic agent from sulphonylureas group, stimulated insulin release through K^+^_ATP_ channel-dependent pathway [43, 52, 53]. This drug acts by closing K^+^_ATP_ channel, depolarizing the plasma membrane, and stimulating the influx of Ca^2+^ through the activation of voltage-dependent calcium channels [54, 55]. In this study, tolbutamide had augmented insulin-releasing effect of 1000 *μ*g/mL of hot aqueous extract indicating that insulin-secretory action of the 1000 *μ*g/mL of hot aqueous extract involved K^+^_ATP_ channel-dependent pathway [34]. IBMX, an inhibitor of phosphodiesterase, raised intracellular cAMP levels of pancreatic *β* cells and enhanced insulin secretion at nonstimulatory glucose concentration [56]. In this study, IBMX did not potentiate insulin releasing effect of 1000 *μ*g/mL of hot aqueous extract suggesting that insulin secretory action of the extract did not involve cAMP production [57], and thus it was not mediated through cAMP-activated pathway such as protein kinase A (PKA) pathway [58].

Diazoxide, an established opener of K^+^_ATP_ channels, inhibited insulin release through activation (opening) of the K^+^_ATP_ channels of the *β* cells [43, 51, 59]. In this study, diazoxide had significantly inhibited insulin-releasing effects of 1000 *μ*g/mL of hot aqueous extract, indicating that activation of K^+^_ATP_ channel participated in the insulin secretory action of the extract. This observation again suggested that K^+^_ATP_ channel-dependent pathway is involved in the insulin secretory action of the extract. It was shown that diazoxide inhibited but did not completely abolish the insulin-releasing effects of 1000 *μ*g/mL hot aqueous extract. This suggested that, beside K^+^_ATP_ channel-dependent pathway, the stimulatory action of the 1000 *μ*g/mL of hot aqueous extract might also involve K^+^_ATP_ channel-independent pathway [60]. This suggestion was supported by observation with 30 mM KCl, a concentration that fully depolarized the cell membrane. Further potentiation of insulin release by 1000 *μ*g/mL of hot aqueous extract under depolarized condition indicating that the extract also exerts insulin-secreting action through K^+^_ATP_ channel-independent manner [50, 51]. In this study, the molecular mechanisms underlying K^+^_ATP_ channel-independent pathway were not evaluated. It was reported that, under K^+^_ATP_ channel-independent pathway, insulin release was triggered through the enhancement of the stimulatory effect of Ca^2+^ on the secretory process [43] or changes adenine nucleotide concentration [42]. Verapamil is an L-type voltage-dependent Ca^2+^ channel (VDCC) blocker from phenylalkylamine classes. This drug inhibited insulin release by preventing Ca^2+^ influx into the cells through L-type VDCC [61]. In this study, verapamil had significantly inhibited the insulin-releasing effects of 1000 *μ*g/mL hot aqueous extract indicating that the insulin secretory action of the extract was dependent on extracellular Ca^2+^ that is acquired through activation of VDCC. It was shown that verapamil only inhibited but did not completely abolish insulin-releasing effects of 1000 *μ*g/mL hot aqueous extract from BRIN BD11 cells indicating that insulin release also occurs in the absence of extracellular Ca^2+^. This suggests that the extract may have the ability to induce mobilization of stored intracellular Ca^2+^ to promote insulin release [34]. The other possible mechanism for ability of the extract to trigger insulin release in the presence of verapamil is the influx of Ca^2+^ occuring through other types of Ca^2+^ channel such as N-type Ca^2+^ channel and voltage-operated Ca^2+^ channel (VOCC), thereby increased intracellular concentration of Ca^2+^ [62–64]. In this study, the effect of *F. deltoidea* extracts on insulin secretion was evaluated at basal glucose concentration (2 mM) only, and no evaluation at high glucose concentration (16.7 mM) was performed. So, it could not be concluded that insulin-secreting activity of *F. deltoidea* extracts is glucose-dependent. It was widely accepted that a good insulinotropic agent must be able to stimulate insulin secretion at both basal and elevated glucose concentrations [65].

Insulin was reported to increase glucose uptake in adipocytes cells [35, 66, 67]. The present study was in accord with such reports that 100 nM insulin significantly increased glucose uptake by approximately 1.64-fold in 3T3F442A adipocyte cells. The same concentration of insulin was also used to stimulate glucose uptake in the presence of *F. deltoidea* extracts. This concentration of insulin was widely used to stimulate glucose uptake into adipocytes cells [68, 69]. In the present study, ethanolic (500 and 1000 *μ*g/mL) and methanolic extracts (500 and 1000 *μ*g/mL) significantly enhance basal (absence of 100 nM insulin) glucose uptake. The enhancement of both extracts at concentration of 1000 *μ*g/mL was significantly higher than that of 100 nM insulin alone indicating that such concentration of extracts enhances glucose uptake in an insulin-mimetic manner. Meanwhile, both extracts at concentrations of 100, 500, and 1000 *μ*g/mL significantly enhance insulin-stimulated (presence of 100 nM insulin) glucose uptake. It was shown that enhancement of 1000 *μ*g/mL of ethanolic extract was significantly higher than that of 100 nM insulin indicating that the extract enhances glucose uptake by sensitizing insulin action [70, 71]. It was shown that 1000 *μ*g/mL of ethanolic extract enhances glucose uptake by both mimicking and sensitizing insulin action. Many antidiabietic plants enhanced glucose uptake through the above mechanisms, for example, *Lagerstroemia speciosa* [35] and *Agaricus campestris* [33] have been reported to enhance glucose uptake in an insulin-mimetic manner, *Salvia miltiorrhiza* Bunge [72] through insulin-sensitizing mechanisms, and *Campsis grandiflora* [73] and *Vaccinium angustifolium* [67] through both mechanisms. Rosiglitazone maleate at concentrations of 35 *μ*M (1.99-fold) and 70 *μ*M (2.27-fold) were used as positive control to challenge glucose uptake activity of *F. deltoidea* extracts under basal and insulin-stimulated states, respectively. These concentrations were chosen because they produced highest enhancement effect after a dose-response evaluation. Rosiglitazone is an antidiabetic drug from thiazolidinedione (TZD) groups. This PPAR gamma agonist has been shown to enhance glucose uptake into adipocytes cells [74]. In this study, 1000 *μ*g/mL of ethanolic and methanolic extracts which evoked an uptake of 2.04- and 1.97-fold, respectively exhibited a comparable potential of basal glucose uptake with 35 *μ*M rosiglitazone maleate. Under insulin-stimulated state, neither ethanolic extract nor methanolic extract exhibited comparable potential of glucose uptake activity with 70 *μ*M rosiglitazone maleate.

Beside stimulation of glucose uptake, insulin also was reported to stimulate adiponectin secretion from adipocytes cells [75, 76]. Treatment of adipocytes cells with 160 nM insulin increased adiponectin secretion by 2-fold [77]. In the present study, 100 nM insulin significantly stimulates adiponectin secretion from 3T3F442A adipocytes cells by 1.60-fold. The hot aqueous extract (500 and 1000 *μ*g/mL) augments basal and insulin-stimulated adiponectin secretion significantly. It was found that basal secretion was higher than the insulin-stimulated secretion, and this suggests that adiponectin secretion activity of hot aqueous extract was independent of insulin. The 500 *μ*g/mL of hot aqueous extract augments basal adiponectin secretion higher than 100 nM insulin indicating that the extract augments adiponectin secretion in an insulin-mimetic manner [78]. Meanwhile, 1000 *μ*g/mL of hot aqueous extract augments both basal and insulin-stimulated adiponectin secretion greater than that of 100 nM insulin suggesting that this extract enhances adiponectin secretion by mimicking and sensitizing insulin action. Methanolic extract at higher concentrations (500 and 1000 *μ*g/mL) augments basal adiponectin secretion significantly whereas lower concentrations (10, 50, and 100 *μ*g/mL) did not affect basal adiponectin secretion. Meanwhile, insulin-stimulated adiponectin secretion was augmented at all concentrations of methanolic extract. Augmentation of adiponectin secretion by methanolic extract was lesser than that of 100 nM insulin alone suggesting that the extract did not mimic or sensitize insulin action. Rosiglitazone maleate was used as positive control to challenge *F. deltoidea* extracts in augmenting adiponectin secretion from 3T3F442A adipocytes. TZD groups have been found to increase expression and synthesis of adiponectin* in vitro* and *in vivo* [79, 80]. In addition, treatment of 3T3-L1 adipocytes with TZDs increased adiponectin secretion by 67–89% [79]. In this study, rosiglitazone maleate increased basal and insulin-stimulated adiponectin secretion by 3.10- and 2.93-fold, respectively. However, neither hot aqueous nor methanolic extracts augmented basal or insulin-stimulated adiponectin secretion to a greater extent than that of rosiglitazone maleate indicating that potential of *F. deltoidea* in augmenting adiponectin secretion was lesser than rosiglitazone maleate. This could possibly be due to that such *F. deltoidea* extracts contain mixture of bioactive and nonbioactive compounds, and there is possibility that the non-bioactive would reduce the concentration of active compounds in the extract and decreased the ability of the extracts to stimulate adiponectin secretion. In contrast, the potential of rosiglitazone maleate which consists of single compound to stimulate adiponectin secretion from adipocytes cells has been scientifically proven [79].

This study had shown hot aqueous, ethanolic, and methanolic extracts of *F. deltoidea* stimulates insulin secretion from BRIN BD11 cells significantly. Nevertheless, insulin-secreting property of ethanolic and methanolic extracts could not be considered because such extracts were found toxic against BRIN BD11 cells. Mechanisms underlying insulin secretory action of hot aqueous extract involve both K^+^_ATP_ channel-dependent and independent pathways. The extract also has the ability to induce the usage of intracellular Ca^2+^ to trigger insulin release. The ethanolic and methanolic extracts have the ability to enhance basal and insulin-stimulated glucose uptake into adipocyte cells. The extracts possess either insulin-mimetic or insulin-sensitizing property or combination of both properties during enhancing glucose uptake into such cells. Ethanolic extract exhibited the higher potential of glucose uptake activity compared to methanolic extract whereas the hot aqueous extract has no effect on glucose uptake activity. Meanwhile, the hot aqueous and methanolic extracts augment basal and insulin-stimulated adiponectin secretion from adipocyte cells. The hot aqueous extract exhibited higher adiponectin secretion activity as compared to methanolic extract whereas the ethanolic extract has no effect on adiponectin secretion activity.

In this study, it was shown that hot aqueous extract exhibited significant insulin secretion and adiponectin secretion activity but has no effect on glucose uptake activity while the ethanolic extract showed significant glucose uptake activity but has no effect on the former two activities. Meanwhile, the methanolic extract exhibited significant adiponectin secretion and glucose uptake activity but not insulin secretion activity. This could be due to the fact that each *F. deltoidea* extract possesses antidiabetic compounds which are unique for some activity only. For example, the hot aqueous extract may own antidiabetic compounds that exert its antidiabetic actions by stimulation of insulin secretion from pancreatic *β* cells and augmentation of adiponectin secretion from adipocytes cells while the ethanolic extract may own antidiabetic compounds that exert its antidiabetic action by enhancement of glucose uptake in adipocytes cells only.

## 5. Conclusions

From this study, it was suggested that the antihyperglycemic actions of *F. deltoidea* are mediated through stimulation of insulin secretion from pancreatic *β* cells, enhancement of glucose uptake by adipocytes cells, and augmentation of adiponectin secretion from adipocytes cells as well. The dual pancreatic and extrapancreatic actions of *F. deltoidea* illustrate the enormous potential of this plant to be developed as new oral antidiabetic drugs. Furthermore, the adiponectin-secreting and insulin-sensitizing properties of *F. deltoidea* indicated that this plant could ameliorate systemic insulin resistance and may potentially be beneficial for type 2 diabetes mellitus related to insulin resistance.

## Figures and Tables

**Table 1:** Effect of **F. deltoidea ** extracts and glibenclamide on BRIN BD11 viability.

*F. deltoidea* extracts	Cell viability (%)					
	Control	10 *μ*g/mL	50 *μ*g/mL	100 *μ*g/mL	500 *μ*g/mL	1000 *μ*g/mL
Hot aqueous	100.00 ± 2.62	95.30 ± 4.37	95.54 ± 4.98	95.66 ± 10.19	99.27 ± 6.19	91.29 ± 2.69 (8.71%)*
Ethanolic	100.00 ± 4.84	92.80 ± 1.54*** (7.20%)	89.60 ± 3.30*** (10.40%)	85.73 ± 1.56*** (14.27%)	38.88 ± 0.94*** (61.12%)	36.46 ± 1.21*** (63.54%)
Methanolic	100.00 ± 6.38	99.32 ± 3.39	97.42 ± 5.62	101.38 ± 2.79	41.80 ± 2.62*** (58.20%)	34.42 ± 0.75*** (65.58%)

	Control	10 *μ*M	100 *μ*M	200 *μ*M	1000 *μ*M	2000 *μ*M

Glibenclamide	100.00 ± 7.24	95.12 ± 7.33	97.46 ± 7.79	97.96 ± 9.03	59.51 ± 12.76*** (40.49%)	32.71 ± 2.79*** (67.29%)

Notes: Cells were incubated for 72 hours in the presence of various concentrations of *F. deltoidea* extracts (10–1000 *μ*g/mL) and glibenclamide (10–2000 *μ*M). Values are expressed as means ± standard deviations (*n* = 8) of percentage of cell viability from three independent assays. *P < 0.05; ***P < 0.001 compared with control. Values between brackets indicate the percentage of cell viability reduction.

**Table 2:** Effect of **F. deltoidea ** extracts and rosiglitazone maleate on 3T3F442A adipocyte viability.

*F. deltoidea* extracts	Cell viability (%)					
	Control	10 *μ*g/mL	50 *μ*g/mL	100 *μ*g/mL	500 *μ*g/mL	1000 *μ*g/mL
Hot aqueous	100.00 ± 9.42	90.75 ± 14.33** (9.25%)	70.59 ± 4.23*** (29.41%)	66.25 ± 2.52*** (33.75%)	55.26 ± 3.51*** (44.74%)	55.03 ± 1.51*** (44.97%)
Ethanolic	100.00 ± 1.10	86.92 ± 3.14*** (13.08%)	72.93 ± 1.34*** (27.07%)	69.60 ± 1.56*** (30.40%)	51.06 ± 0.60*** (48.94%)	55.07 ± 6.14*** (44.93%)
Methanolic	100.00 ± 1.10	88.65 ± 3.42*** (11.35%)	72.54 ± 1.45*** (27.46%)	70.99 ± 1.65*** (29.01%)	62.61 ± 4.01*** (37.39%)	51.55 ± 1.00*** (48.45%)

	Control	3.5 *μ*M	7 *μ*M	35 *μ*M	70 *μ*M	140 *μ*M

Rosiglitazone maleate	100.00 ± 7.05	91.57 ± 6.58	77.10 ± 7.31*** (22.90%)	71.97 ± 6.53*** (28.03%)	54.01 ± 7.41*** (45.99%)	49.51 ± 5.74*** (50.49%)

Notes: Cells were incubated for 72 hours in the presence of various concentrations of *F. deltoidea* extracts (10–1000 *μ*g/mL) and rosiglitazone maleate (3.5–140 *μ*M). Values expressed as percentage of means ± standard deviations (*n* = 8) of the cells viability from three independent assays. **P < 0.01; ***P < 0.001 compared with control. Values between brackets indicate percentage of cell viability reduction.

**Table 3:** Effect of *F. deltoidea* extracts on glucose uptake in 3T3F442A adipocytes.

	Fold of glucose uptake against control									
Extract concentration (*μ*g/mL)	Control	100 nM insulin	Hot aqueous		Ethanolic		Methanolic		Rosiglitazone malate	
			Basal	Insulin-mediated	Basal	Insulin-mediated	Basal	Insulin-mediated	Basal	Insulin-mediated
10			0.88	1.00	1.11	1.24	1.02	1.16	35 *Μ*m 1.99***	70 *μ*M 2.27***
50			0.86	0.92	1.14	1.22	1.15	1.18		
100	1.00	1.55***	1.13	1.45	1.18	1.35*	1.32	1.25*		
500			1.49	1.29	1.61***	1.69***	1.71 ***	1.54***		
1000			1.28	1.32	2.04*** ••	1.99*** •	1.97*** •	1.68***		

Values expressed as means ± standard deviations from three independent experiments.

*P < 0.05, ***P < 0.001 compared with control incubation.

^•^ *P* < 0.05, ^••^ *P* < 0.01 compared to 100 nM insulin.

**Table 4:** Effect of *F. deltoidea* extracts on adiponectin secretion from 3T3F442A adipocytes.

	Fold of glucose uptake against control									
Extract concentration (*μ*g/mL)	Control	100 nM insulin	Hot aqueous		Ethanolic		Methanolic		Rosiglitazone malate	
			Basal	Insulin-mediated	Basal	Insulin-mediated	Basal	Insulin-mediated	Basal	Insulin-mediated
10			1.01	0.93	1.06	0.99	1.11	1.45***	70 *μ*M 3.10*** •••	3.5 *μ*M 2.93*** •••
50			1.16	1.05	0.99	1.04	1.09	1.46***		
100	1.00	1.64***	1.20	1.21	1.04	1.12	1.28	1.53***		
500			2.06*** •••	1.48***	1.05	1.15	1.30*	1.67***		
1000			2.46*** •••	2.10*** •••	1.17	1.24	1.53***	1.70***		

Values expressed as means ± standard deviations from three independent experiments.

*P < 0.05, ***P < 0.001 compared with control incubation.

^•••^ *P* < 0.001 compared to 100 nM insulin.
